# Effects of Zilpaterol Hydrochloride with a Combination of Vitamin D_3_ on Feedlot Lambs: Growth Performance, Dietary Energetics, Carcass Traits, and Meat Quality

**DOI:** 10.3390/ani14091303

**Published:** 2024-04-26

**Authors:** Karla H. Leyva-Medina, Horacio Dávila-Ramos, Jesús J. Portillo-Loera, Omar S. Acuña-Meléndez, Adriana Cervantes-Noriega, Jaime N. Sánchez-Pérez, Gamaliel Molina-Gámez, Javier G. Rodríguez-Carpena, Mario A. Mejía-Delgadillo, Juan C. Robles-Estrada

**Affiliations:** 1 Faculty of Veterinary Medicine and Zootechnics, Autonomous University of Sinaloa, Culiacan 80246, Sinaloa, Mexico; karla-hlm@hotmail.com (K.H.L.-M.); davila-ramos@uas.edu.mx (H.D.-R.); portillo6422@uas.edu.mx (J.J.P.-L.); osacunam@uas.edu.mx (O.S.A.-M.); adrianacern@gmail.com (A.C.-N.); jaime.sanchez.fmvz@uas.edu.mx (J.N.S.-P.); gamalielmolinagamez@gmail.com (G.M.-G.); 2 Academic Unit of Veterinary Medicine and Zootechnics, Autonomous University of Nayarit, Compostela 63700, Nayarit, Mexico; german.rc@uan.edu.mx; 3 Faculty of Agronomy, Autonomous University of Sinaloa, Culiacan 80000, Sinaloa, Mexico; mamdz@uas.edu.mx

**Keywords:** zilpaterol hydrochloride, vitamin D3, ruminant, growth performance, carcass traits, meat quality

## Abstract

**Simple Summary:**

Zilpaterol hydrochloride (ZH) is an additive used to enhance the growth performance and dressing carcass in ruminants. However, its use can lead to a reduction in meat quality, resulting in tougher meat. Meat tenderness is a crucial quality trait that affects consumer acceptability, satisfaction, and repeat purchase. Studies have shown that supplementing ruminants with vitamin D_3_ (D3) before slaughter can improve meat tenderness. It is believed that supplementing cattle treated with ZH with D3 can mitigate the negative effects of ZH and improve meat quality. In this study, we aimed to evaluate the combined effect of ZH and D3 on growth performance, energy efficiency, carcass traits, and meat quality of feedlot lambs. ZH supplementation improved growth, energy efficiency, and carcass quality but resulted in tougher meat. In contrast, supplementing lambs with D3 negatively affected dry matter intake and weight gain. However, D3 improved meat pH and color attributes but had no effect on reducing toughness. In addition, lambs fed both additives (ZH and D3) as well reduced their dry matter intake and weight gain. The inclusion of vitamin D did not reduce the toughness of the meat related to the use of zilpaterol.

**Abstract:**

This study evaluated the impact of supplementing ZH in combination with D3 on the growth performance, energy efficiency, carcass traits, and meat quality of feedlot lambs. Thirty-two Dorper × Katahdin cross lambs (37.3 ± 5.72 kg) were utilized in a 29 d experiment in a completely randomized block design with a 2 × 2 factorial structure consisting of two levels of ZH for 26 d (0 and 0.20 mg/kg PV^−1^) and two levels of D3 for 7 d (0 and 1.5 × 10^6^ IU/d^−1^). ZH improved (*p* ≤ 0.05) the average daily gain (ADG) and feed efficiency by 9.9% and 17.8%, respectively, as well as hot carcass weight (HCW) and dressing carcass by 4.3% and 2.6%, respectively. (*p* ≤ 0.03). However, ZH increased (*p* < 0.01) muscle pH and Warner–Bratzler shear force (WBSF) (2.5 and 23.0%, respectively). D3 supplementation negatively affected (*p* ≤ 0.02) dry matter intake (DMI) (last 7 d) and ADG by 15.7% and 18.1%. On the other hand, D3 improved the pH of the longissimus thoracis muscle by 1.7% (*p* = 0.03) without affecting WBSF. When D3 was supplemented in combination with ZH, it was observed that meat quality was improved by reducing muscle pH compared to lambs treated only with ZH. However, D3 did not improve the meat tenderness negatively affected by ZH supplementation.

## 1. Introduction

Beta-agonists are commonly used in beef production to increase dressing carcass weight and cutability by promoting muscle protein synthesis and reducing body and carcass fat [1]. Zilpaterol hydrochloride is a β_2_-adrenergic agonist feed additive whose effect is not permanent, and its benefits are obtained between 20 and 30 d of supplementation before slaughter, at a daily dose of approximately 0.15 mg/kg of live weight. In lambs, it has been observed that daily doses of up to 0.20 mg/kg of live weight show a better response compared to cattle [2,3]. This additive has been approved for use in beef feedlots in South Africa since 1995, Mexico since 1996, and the USA since 2006 [4]. However, countries, such as those of the European Union (EU) and Russia, have legislation that ban the importation of products that were produced using β-adrenergic agonists [5]. Studies have shown that the use of ZH can have a negative impact on meat quality by increasing the meat toughness measurements [6,7,8,9]. Several factors can contribute to an increase in WBSF values resulting from ZH supplementation. One of the reasons is alterations to the activity of the myofibrillar enzyme system, which may occur due to a decrease in calpain concentrations and an increase in the activity of calpastatin [10,11]. Likewise, previous studies indicate the incidence of hypertrophy, an increase in muscle fiber diameter [12], and a decrease in intramuscular fat increase WBSF [13]. Meat tenderness is a major eating quality attribute that ensures consumer satisfaction and repeat purchase of red meat. The variability in meat tenderness is related to several factors that are spread across the production chain in fresh red meat products [14]. Different strategies have been implemented to improve this characteristic with variable effectiveness. The addition of D3 to feed for a short period of time prior to slaughter is one of the alternatives that have demonstrated improvements in meat tenderness [15]. D3 supplemented to feed before slaughter increases intramuscular calcium concentration [16,17,18,19] and activates calpain enzymes that degrade myofibrillar proteins [20], leading to improved meat tenderness [21,22,23]. Therefore, oral supplementation with supra-nutritional doses of D3 seems to be an alternative means of enhancing meat tenderness in cattle treated with ZH [19]. Based on this, we hypothesized that supplemental D3 and ZH in feedlot lambs could improve growth performance, carcass traits, and meat quality characteristics by reducing WBSF. Therefore, the objective of this experiment was to determine the effect of the addition of D3 in diets with ZH in feedlot lambs on growth performance, dietary energetics, carcass characteristics, and meat quality.

## 2. Materials and Methods

### 2.1. Animal Care, Handling and Facilities

This study was carried out in the Experimental Growth Unit for Small Ruminants at the Faculty of Veterinary Medicine and Zootechnics of the Autonomous University of Sinaloa (24.7721 N, −107.3545 W). All live animal handling was conducted in accordance with the Official Mexican norms for animal care [24,25,26] and of the Institutional Committee for the Care and Use of Animals of the Veterinary Medicine and Animal Science Faculty (CICUA-FMVZ/17-10-2016).

Four weeks before the beginning of the experiment, forty-two lambs were adapted to the management, facilities, experimental diet and treated against parasites (Saguaymic plus, Microsules Lab, Mexico city, Mexico), hemoparasites (Imizol, Merck & Co., Rahway, NJ, USA) and injected with 1 × 10^6^ IU of vitamin A (Synt-ADE, Fort Dodge, Animal Health, Mexico city, Mexico). Any lambs that showed illness or atypical growth performance were excluded.

From the remaining group, thirty-two Dorper × Katahdin cross intact male lambs with an average initial live weight of 37.3 ± 5.72 kg and aged 5 months were utilized in a 29 d experiment, incorporating pre-slaughter supplementation of two levels of ZH over 26 d plus a 3 d withdrawal period (0 and 0.20 mg/kg LW^−1^) and two levels of D3 over 7 d (1.5 × 10^6^ IU/lamb/d^−1^). ZH (ZILMAX, Merck & Co., Rahway, NJ, USA) was provided at a total dose of approximately 9 mg/lamb daily for 26 d, followed by a three-day withdrawal period. Additionally, D3 (Microvit D3, Adisseo lab, Alpharetta, GA, USA) was introduced to feed during the final 7 d leading up to slaughter. Lambs were blocked by live weight into four uniform weight groups and assigned to 16 pens (2 lambs/pen and a total of 8 lambs per treatment). Dietary treatments were randomly assigned to pens within blocks, with four replicates per treatment. Pens have 6 m^2^ with overhead shade, 1 m fence-line feed bunks, and a manual waterer with ad libitum access.

### 2.2. Experimental Diet

The diet was formulated based on cracked corn and soybean meal, with a crude protein content of 14.5% and 1.43 Mcal/NEg (Table 1), based on the nutritional value of the ingredients, as outlined in nutrient requirements for sheep tables [27]. It was provided twice a day, with a morning-to-afternoon service percentage of total feed intake of 30:70 (0900 and 1500 h). Before the morning feed, the bunk feeders were checked 30 min in advance to assess the previous day’s intake. Any excess feed was removed, weighed, and recorded. This information was then used to adjust the afternoon feed to ensure a refusal rate of less than 7.0%.

### 2.3. Calculations

Feed samples were collected daily for dry matter analysis [28]. To evaluate the impact of treatments on growth performance, lambs were weighed at the start and end of the experiment. Moreover, to assess the effect of the treatments on carcass performance expressed as ADG and feed efficiency, the final live weight (FLW) adjusted to carcass weight was calculated as HCW/overall average of dressing carcass (55.5%) for all treatments. Assuming that DMI is related to energy requirements and dietary NEm, it is expected that the DMI can be estimated from average ADG and LW values according to the following equation: $\mathrm{DMI},\;\mathrm{kg}/d\;=\left(\frac{\mathrm{ME}}{2.06}\right)+\left(\frac{\mathrm{EG}}{1.40}\right)$, where ME (energy required for maintenance, Mcal/d) = $\mathrm{ME}=56\times {\mathrm{SBW}}^{0.75}$, SBW, kg = (shrunk body weight, ${\mathrm{BW}}^{0.96}$), EG (energy gain, Mcal/d) = $276\times \mathrm{ADG}\times {\mathrm{SBW}}^{0.75}$ and NEm and NEg are 2.06 and 1.40 Mcal/kg, respectively, derived from tabular values based on ingredient composition of the experimental diet [27]. A coefficient of 276 was estimated assuming a mature weight for Pelibuey × Katahdin male lambs of 115 kg [27]. Dietary net energy was estimated utilizing the quadratic formula [29], $x=\frac{-b\;-\;\sqrt{b2\;-\;4\mathrm{ac}}}{2c}$, where: x = Nem, a = −0.41, EM, b = 0.877 EM + 0.41 DMI + EG, and c = −0.877 DMI.

### 2.4. Carcass Traits and Primary Cuts

Three days before the processing of the lambs at the slaughterhouse, ZH supplementation was removed from the diets. D3 was withdrawn from the diets 24 h before slaughter, which corresponds to the fasting time before transport to the slaughterhouse. After completing the growth test, the animals’ FLW was measured, and then the animals were transported in a small ruminant livestock trailer to the slaughterhouse facilities to obtain the carcass. Upon arrival, they were unloaded in a lairage pen, where they had access only to drinking water.

After the animals were slaughtered, the HCW was measured, and the percentage of the dressing carcass was subsequently calculated. The carcasses were stored in a cold room (2 °C) for 24 h before being transported to the meat cutting room. The carcasses were divided longitudinally by the center of the vertebrae, and the thickness of back fat was subsequently recorded with a digital vernier caliper (ModelA020, Cadena, Mexico) measured at a point ¾ of the length of the longissimus dorsi muscle from the split chine bone and the area of longissimus thoracis muscle (LTM) using a grid plastic device positioned in the cross-section (between 12 and 13th rib) of the left half or the carcass. To determine the quantity of perirenal and pelvic fat, it was manually removed from the carcass, and the weight was recorded and subsequently expressed as a percentage of cold carcass weight. The half carcasses were divided into primary cuts, their weight was recorded, and the percentage of each cut was calculated with respect to cold carcass weight. Primary cuts were obtained in accordance with the guidelines of the North American Meat Processors Association (NAMP) [30]. The front quarter of the carcass was divided into the following: neck, foreshank (208D), shoulder (207), rib (209A), rack (204), and breast (209); the hind quarters were divided into the following: flank (232E), loin (232A), and leg (233A) [30].

### 2.5. Meat Quality and Fatty Acid Profile

The LTM was obtained from the Rack-204 primary cut of the loin of all lambs; after removing the bone and fat, it was then divided into two samples and packaged under vacuum; a (1) 50 g sample of the distal portion (−20 °C) was taken for the determination of the fatty acid profile; and (2) a sample of the remaining muscle (~500 g) was stored for 7 d at 2 °C, subsequently frozen and transported for the determination of the meat quality characteristics. 

The sample for meat quality determination was defrosted at 4 °C for 24 h. For the pH analysis, a portable pH meter with a puncture electrode was used (Delta track-ISFET pH101, Pleasanton, CA, USA). The color was expressed according to the system of the International Commission on Illumination CIELAB color space and reported as L* (lightness), a* (redness), and b* (yellowness), and the measurements were taken using a Konica Minolta CR-10 reader (Ramsey, NJ, USA). The calibration of the instrument was carried out before the measurement by placing the CR-10 reader against white and black tiles. To determine the tenderness, the meat sample was placed in a high-temperature-resistant plastic bag and subsequently placed in a water bath at 75 °C for 1 h. Cores were taken (1 × 1 × 3 cm) from each cooked steak, parallel to the orientation of the muscle fibers. The WBSF measurements were determined using a texturometer (Lloyd Instruments, Hampshire, UK) equipped with Warner–Bratzler shear blades with a crosshead speed of 50 mm/min.

The water holding capacity (WHC) was determined following the procedure described by [31], which is based on the capacity of meat samples (1.5 cm × 1.5 cm) to retain water after centrifugation (3600× *g* at 4 °C for 5 min). The WHC percentage was calculated based on the difference in weight of the sample before and after centrifugation. 

For the determination of fatty acids, 500 mg samples of dry meat (Lyophilizer Ilshin Biobase FD-8508, Roshtec, Bengaluru, India) were deposited in Pyrex tubes with teflon stoppers. To each sample, 2 mL of hexane and 3 mL or 5% methanolic hydrochloric acid were added, and the mixture was shaken carefully for 1 min to homogenize the sample. It was then placed in a 70 °C water bath for 45 min. After cooling for 20 min at room temperature, 5 mL of 6% potassium carbonate and 1 mL of hexane were added. The samples were then shaken for 1 min and centrifuged at 1500 rev min^−1^ (200 g) for 5 min. The supernatant was then immediately transferred to sterile Pyrex tubes containing 1 g of sodium sulfate. Again, the samples were shaken for 1 min and centrifuged for 5 min at 1500 rev min^−1^ (200 g) to finally extract the supernatant with Whatman (grade 597) filter paper. These samples were put into Eppendorf vials and stored at −40 °C until analysis. Under working conditions, the chromatograph (Perkin Elmer, model Clarus 500, Waltham, MA, USA) was supplied with a capillary column with the dimensions of 100 m × 0.25 mm × 0.2 μm (SUPELCO TM-2560, Sigma–Aldrich, St. Louis, MI, USA); nitrogen carrier gas was used, and the oven temperature was maintained at 140 °C for 5 min with increases of 4 °C per min up to 240 °C. The injector and detector were maintained at 260 °C. The peaks were identified according to the retention times of the methyl ester standards (SUPELCO37, FAME MIX analytical Sigma–Aldrich, St. Louis, MI, USA).

### 2.6. Statistical Analysis

The following data were analyzed as part of a randomized complete block design: growth performance, dietary energetics, carcass traits, and primary cuts components. The meat quality component data were analyzed as part of a completely randomized design. The experiment used a factorial arrangement of 2 × 2, with two levels of ZH (0 and 0.20 mg/kg LW d^−1^) and two levels of D3 (0 and 1.5 × 10^6^ IU/lamb^−1^), considering a pen as the experimental unit. The SAS program’s MIXED procedure was used to analyze the variables (SAS Inst. Inc., Cary, NC, USA). The fixed effect was based on the treatments, while the pen was considered a random component. Treatment effects were tested as follows: (a) ZH level inclusion, (b) D3 level inclusion, and (c) ZH × D3 interaction. To observe interaction responses, a comparison of multiple means was carried out using the Tukey test on ADG, feed efficiency, dietary net energy, HCW, meat redness (a*), pH meat, WBSF, and fatty acids variables. For a comparison of treatment means (all figures), the following abbreviations were used: zilpaterol (ZIL), vitamin D3 (VIT), and zilpaterol plus vitamin D3 (ZIL+VIT). The analyses were considered significant if the *p*-value was ≤0.05 and showed a tendency if the probability value was *p* ≤ 0.1.

## 3. Results

### 3.1. Growth Performance

The main effects of ZH and D3 on the growth performance variables in lambs are shown in Table 2. Supplementation with ZH did not alter the average FLW and DMI during the entire trial or DMI during the last 7 d of the trial. However, ZH reduced DMI as a percentage of the average LW by 6.5% (*p* = 0.04). Likewise, ADG, total weight gain, and feed efficiency experienced an improvement of (*p* = 0.05) 9.9, 9.8, and 17.8%, respectively. When analyzing growth performance components considering the FLW adjusted-carcass, lambs treated with ZH showed significant improvement (*p* < 0.01) in FLW, ADG, and feed efficiency (4.3, 28.2, and 36.8%, respectively).

Vitamin D_3_ supplementation did not modify DMI overall. However, during the last 7 d of the experiment, DMI was reduced by 15.7% (*p* = 0.02) due to D3 supplementation. Additionally, the use of D3 resulted (*p* = 0.04) in a 2.7% reduction in FLW, and ADG and total LW gain were also negatively affected by 18.1% and 18.0%, respectively (*p* < 0.01). As a result, the combination of lower LW gains and DMI reduction led to a decrease (*p* < 0.01) in feed efficiency by 25.9%. While D3 supplementation did not impact the FLW carcass-adjusted value, it did negatively affect ADG and feed efficiency carcass-adjusted values by 17.0% and 25.5%, respectively (*p* < 0.01).

**Figure 1:**
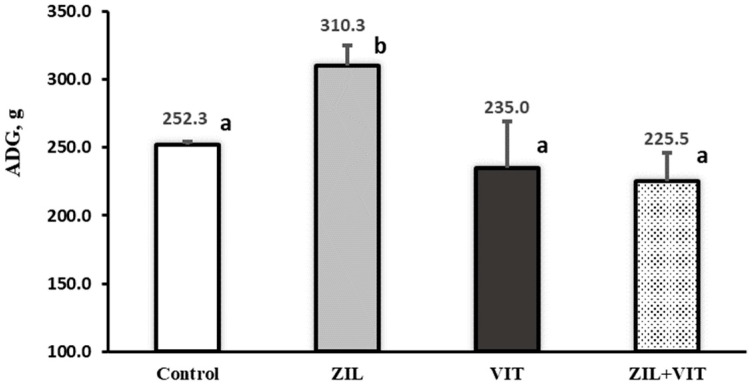
The figure shows the effect of zilpaterol and vitamin D_3_ on ADG of feedlot lambs. Values with different letters are significantly different (*p* ≤ 0.05).

**Figure 2:**
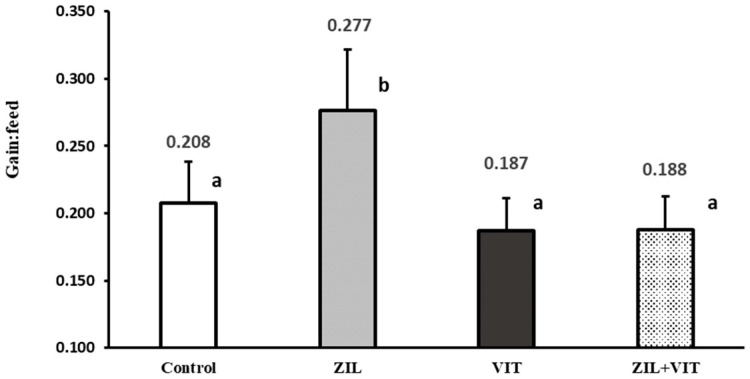
The figure shows the effect of zilpaterol and vitamin D_3_ on the feed efficiency of feedlot lambs. Values with different letters are significantly different (*p* ≤ 0.05).

The ZH treatment was shown to have significant interaction with D3 in terms of growth performance variables (*p* ≤ 0.03), such as ADG, total LW gain, and feed efficiency. Likewise, those variables calculated using the FLW carcass-adjusted values showed an interaction between ZH and D3 (*p* < 0.01). The effects of the treatments on ADG and feed efficiency are shown in Figure 1 and Figure 2. ZIL supplementation improved ADG by 22.8% (*p* < 0.01) compared to the control group. However, lambs treated with ZIL+VIT showed a similar performance to the control group. Similarly, feed efficiency was enhanced by 33.1% (*p* < 0.01) when using ZIL supplementation, but this positive effect was nullified when supplemented with ZIL+VIT.

### 3.2. Dietary Energetics

**Figure 3:**
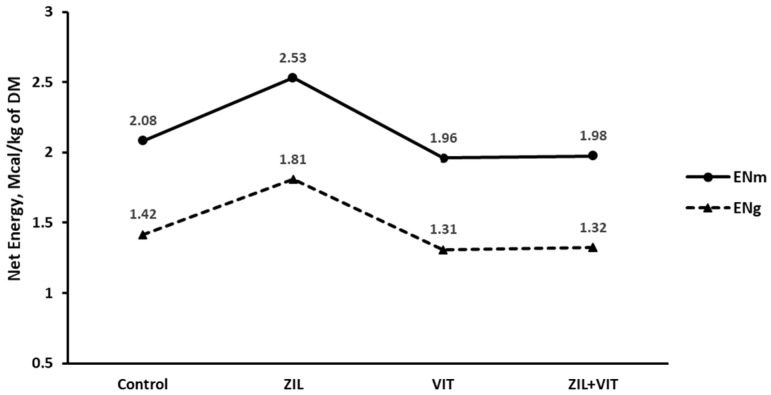
The figure shows the effect of treatments on the dietary energetics of feedlot lambs.

Table 3 outlines the main effects of the dietary energetics of feedlot lambs. During the trial, dietary energetics improved with the use of ZH supplementation, as determined by LW gain and DMI. ZH significantly improved dietary NEm and NEg by 11.3% and 14.7%, respectively (*p* < 0.01). These improvements are reflected in the observed/expected ratio for DMI, NEm, and NEg, which increased by 11.3%, 11.4%, and 14.7%, respectively (*p* ≤ 0.01). The inclusion of D3 in the diet of lambs for 7 d prior to slaughter had an adverse effect on the calculation of dietary energy derived from LW gain and DMI during the experiment. This resulted in a significant reduction (*p* < 0.01) in NEm and NEg by 14.7% and 18.6%, respectively. Additionally, there was a decrease (*p* < 0.01) of 20.0% in the efficiency of the observed/expected DMI ratio. All of the calculations used to estimate dietary energy from LW gain and DMI showed (Figure 3) a negative interaction response (*p* ≤ 0.02). Despite the positive response in terms of growth performance components, the addition of ZIL+VIT to the diet did not improve energy efficiency.

### 3.3. Carcass Traits and Primary Cuts

**Figure 4:**
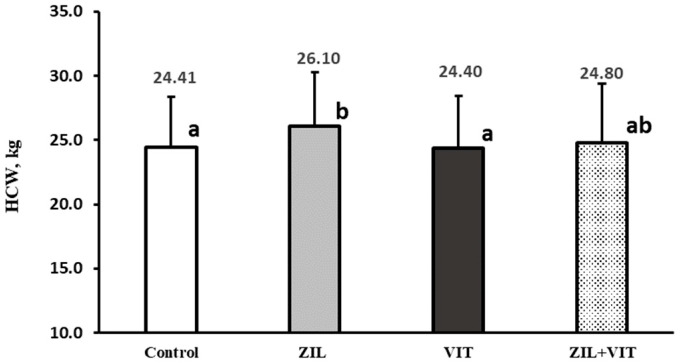
The figure shows the effect of treatments on hot carcass weight of feedlot lambs. Values with different letters are significantly different (*p* ≤ 0.05).

The results of the treatments on the carcass traits are shown in Table 4. ZH administration for 26 d followed by a 3 d withdrawal period resulted in significant improvements (*p* ≤ 0.03) in terms of HCW, dressing percentage, and LTM area by 4.3%, 2.6%, and 6.7%, respectively. However, adipose tissue characteristics, such as fat thickness and perirenal–pelvis fat, remained unchanged. On the other hand, D3 did not affect dressing carcass percentage, LTM area, fat thickness, or perirenal–pelvis fat percentage. However, there was a tendency (*p* = 0.07) for D3 to negatively impact HCW by 2.54%. Additionally, there was a trend (*p* = 0.07) of interaction between ZIL+VIT, resulting in negative effects on HCW (Figure 4). The primary meat cuts components (Table 5) were not impacted by either the ZH (*p* > 0.35) or D3 (*p* > 0.27) treatments. Furthermore, no interactions between the treatments were identified (*p* > 0.13).

### 3.4. Meat Quality and Fatty Acid Profile

The main effects of the treatments on the meat quality characteristics are shown in Table 6. ZH supplementation resulted in significant changes in meat quality, reducing (*p* = 0.02) lightness (L*) and WHC by 7.0 and 3.4%, respectively, compared to untreated lambs. Conversely, ZH increased (*p* < 0.01) meat pH and WBSF by 2.5% and 21.5%, respectively, and 23.8%, respectively.

ZH did not impact the intensity of redness (a*) or yellowness (b*). The addition of D3 supplementation improved the meat color characteristics, increasing (*p* ≤ 0.02) the intensity of lightness (L*), redness (a*), and yellowness (b*) by 8.0%, 6.7%, and 17.0%, respectively. Additionally, the use of D3 led to a (*p* = 0.03) pH drop in meat by 1.7% compared to the untreated lambs but did not affect the WBSF values. However, D3 supplementation reduced WHC by 2.9% compared to non-supplemented lambs.

**Figure 5:**
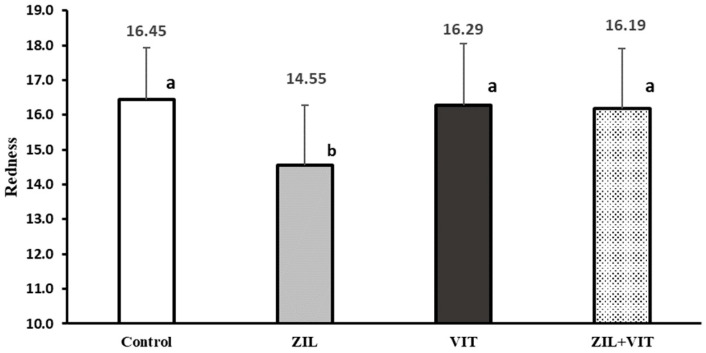
The figure shows the effect of treatments on the redness (a*) of longissimus dorsi of feedlot lambs. Values with different letters are significantly different (*p* ≤ 0.05).

**Figure 6:**
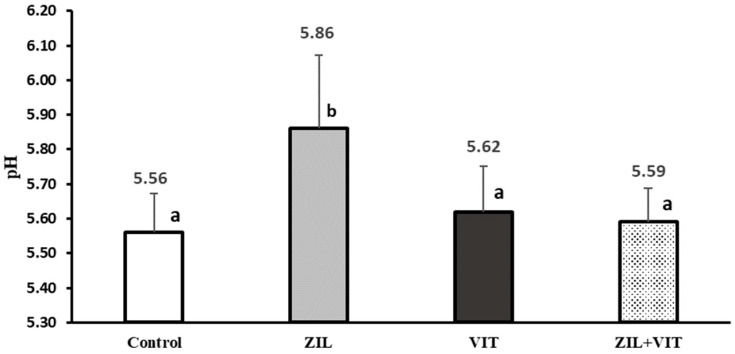
The figure shows the effect of treatments on the pH of longissimus dorsi of feedlot lambs. Values with different letters are significantly different (*p* ≤ 0.05).

**Figure 7:**
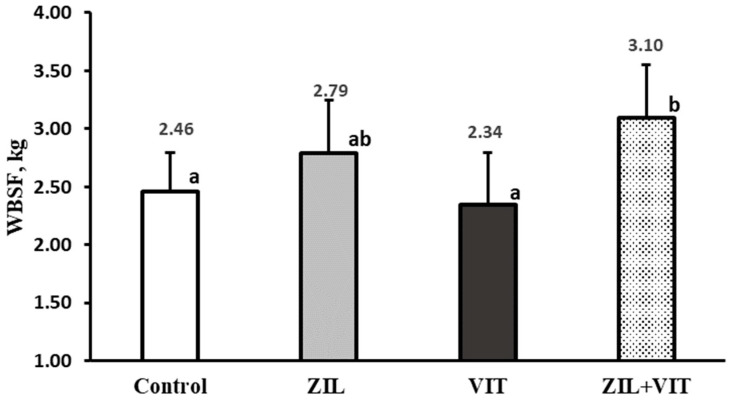
The figure shows the effect of treatments on WBSF of longissimus muscle. Values with different letters are significantly different (*p* ≤ 0.05).

In Figure 5 and Figure 6, an interaction (*p* < 0.01) between the variables of redness intensity (a*) and meat pH with the combined supplementation of ZIL+VIT is observed. ZIL treatment had a negative effect (*p* < 0.01) on the intensity of redness, decreasing it by 11.5% compared to the control group. However, when combined with VIT, the redness intensity of lambs treated with ZIL+VIT improved by 11.2% compared with ZIL; however, there was no significant difference compared to other treatments. Similarly, ZH maintained high meat pH values (*p* < 0.01) concerning the control, ZIL, and VIT treatments, and when comparing ZIL to the control group, the pH was 5.3% higher. However, when supplementing ZIL+VIT, the final pH of the meat decreased by 4.6% when compared to lambs treated with ZIL alone. Finally, lambs treated with ZIL+VIT had a similar final pH to the control and VIT groups. Additionally, there was an interaction between ZIL and VIT in the muscle’s WBSF. Contrary to expectations, the ZIL+VIT treatment led to higher WBSF values than the control and VIT treatments, indicating an increase in meat toughness (Figure 7).

The main effects of the treatments on the fatty acid profile of the intramuscular fat of LTM are shown in Table 7. Lambs treated with ZH showed a 19.4% reduction in the percentage of palmitoleic fatty acid in intramuscular fat compared to untreated lambs (*p* = 0.10). However, no significant changes were observed in the rest of the analyzed fatty acids due to treatment with ZH. On the other hand, supplementation with D3 resulted in a reduction (*p* = 0.05) of 51.8% in the percentage of linolenic fatty acid in the samples analyzed. Additionally, there was a trend (*p* = 0.10) towards a 5.7% reduction in oleic fatty acid in the intramuscular fat of lambs treated with D3 compared to untreated ones.

**Figure 8:**
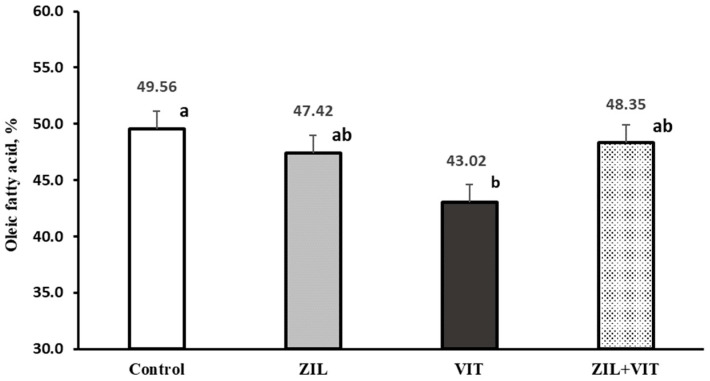
The figure shows the effect of treatments on oleic fatty acid taken from longissimus of feedlot lambs. Values with different letters are significantly different (*p* ≤ 0.05).

**Figure 9:**
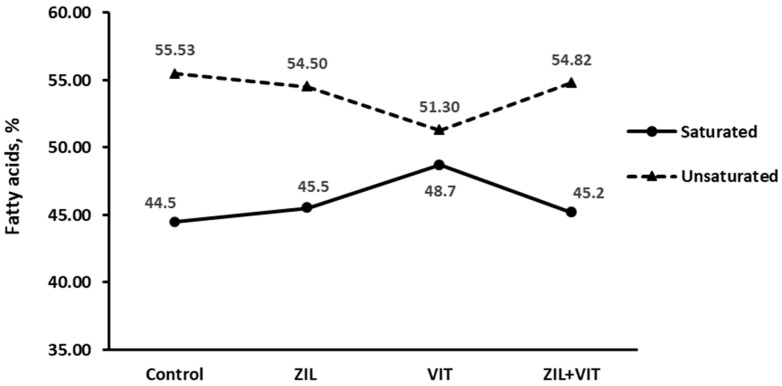
The figure shows the effect of treatments on fatty acids taken from longissimus of feedlot lambs.

An interaction response (*p* = 0.03) between ZIL and VIT was observed in the percentage of oleic fatty acid of intramuscular fat. Figure 8 shows that the treatment using VIT reduced the percentage of oleic fatty acid in intramuscular fat by 13.1% compared to the control group. However, this effect did not occur when supplemented with ZIL or ZIL+VIT. It was observed that there was an interaction trend (*p* = 0.09) with ZIL+VIT supplementation. Lambs who were supplemented with VIT experienced a decrease in unsaturated fatty acids and an increase in saturated fatty acids, as shown in Figure 9. However, these effects were not observed in the samples analyzed from lambs who were supplemented with ZIL+VIT.

## 4. Discussion

### 4.1. Growth Performance, Energetics, and Carcass Traits

According to [32], the primary impact of ZH on ruminant production is linked to the enhancement of both LW and the dressing carcass. This is attributed to an increase in muscle protein synthesis and retention, which occurs concurrently with the suppression of lipogenesis and an increase in lipolysis. ZH activates a biological mechanism that improves growth performance indicators and energy retention due to higher muscle tissue concentration compared to adipose tissue. This is confirmed by calculations that estimate the energy intake of the diet [27,29], indicating an apparent increase in the NEm, NEg, and DMI observed/expected ratios. Multiple studies have demonstrated the effectiveness of the use of ZH in lamb production. For instance, research conducted with ZH in lambs with LW, doses, and results similar to those of the present study, showed improvements in growth indicators [33,34,35,36]. These studies have reported an increase in ADG, feed efficiency, and energy retention provided by the diet (12.5–25.0%, 13.0–36.0%, and 9.5–35.0%, respectively). Similarly, the results on carcass quality traits from some authors [34,37,38] coincide with those presented in our study. These studies indicate that the effects of ZH supplementation are observed in relation to carcass quality, such as an increase in the HCW (7.0–11.2%), dressing carcass (2.0–8.8%), LTM area (11.2–15.4%), and a reduction in perirenal–pelvic fat (21.7–26.1%).

Vitamin D_3_ acts as a precursor of 1,25-dihydroxycholecalciferol, a hormone involved in calcium and phosphorus homeostasis [39]. Previous studies have shown that feeding supra-nutritional doses of D3 in ruminants can cause hypercalcemia and reduced DMI and body weight [22]. In a study by [22], steers were supplemented with 5 × 10^6^ IU of D3 for 10 d during the ZH supplementation stage (21 d), resulting in a significant decrease in ADG, DMI, and feed efficiency by 47.6%, 11.5%, and 52.0%, respectively. Similarly, [16] reductions in ADG and feed efficiency of up to 25.1% and 23.0% were observed, respectively, when testing the supplementation of 5 × 10^6^ IU of D3 in steers for 24 d. Our findings align with these results, where administering D3 led to an 18.1% reduction in ADG and a 25.9% reduction in feed efficiency over a period of 29 d, although the overall DMI was not affected. However, DMI during the last 7 d was reduced by 15.7%.

Studies with supra-nutritional doses of D3 supplementation were carried out in short periods before slaughter. This approach should ensure that the carcass quality is not compromised by DMI reduction due to D3 supplementation. However, some studies suggest that there may be a slight impact on the quality of the carcass. Studies have shown that supplementing D3 in steers and lambs before slaughter can result in a significant reduction in weight and fat thickness. Authors [22] found that supplementing 5 × 10^6^ IU of D3 for 10 d led to a reduction in HCW and back fat thickness by 3.8% and 16.3%, respectively. Studies [40] reported that supplementing 6 million IU of D3 for 4–6 d prior to slaughter resulted in a reduction in FLW by 4.7%, HCW by 5.3%, and back fat thickness by 19.1%. Similarly, [41] observed a 19.0% reduction in adjusted fat thickness in lambs when supplementing 7.5 × 10^6^ IU of D3 for 4 d before slaughter, without any changes in dressing carcass and LTM area.

On the contrary, a study conducted by [16] observed a 20.2% increase in back fat thickness in steer carcasses when feeding 5 × 10^6^ IU of D3, without affecting HCW and dressing carcass. However, providing high doses of D3 may lead to a reduction in DMI, which can reduce the supply of nutrients for body maintenance and result in catabolism of fat tissue. This can ultimately lead to a reduction in the USDA yield grade. Nonetheless, other studies [16] did not show this pattern of results when administering D3. Although the negative effect of D3 on DMI, ADG, and feed efficiency is an expected response, ZH supplementation could compensate for this effect. The negative interaction is the result of the inability of ZH to compensate for the reduction in DMI. It seems that ZH cannot have a favorable response in growth performance when the energy available in the diet is reduced due to low DMI.

### 4.2. Meat Quality

Meat tenderness is a crucial quality trait that affects consumer acceptability, satisfaction, and repeat purchase [42]. Muscle hypertrophy brought about by ZH supplementation consequently increases the toughness of the meat. Various factors can contribute to an increase in WBSF values due to ZH supplementation. One reason for this is the alteration to the activity of the myofibrillar enzyme system, which may occur due to decreased calpain concentrations and increased calpastatin activity [10,18,43]. Research has also shown that an increase in muscle fiber diameter and hypertrophy [12] and a decrease in intramuscular fat levels [13] can raise WBSF values. The calpain–calpastatin proteolytic system has been identified as the key factor that determines meat tenderness.

However, some authors suggest that aging can help mitigate the negative impact of meat toughness [44]. The effectiveness of the aging strategy in improving the WBSF of muscles treated with ZH is not consistent. ZH has been shown to increase WBSF in cattle, which has been widely documented [8]. Supplementing with ZH for 20 d resulted in tougher meat (7.0–21.4%) in the LTM across different periods of aging. Other studies [45,46] have reported similar increases in WBSF (8.8–29.8%) in the LTM with ZH supplementation ranging from 20 to 40 d. The impact of ZH on muscle WBSF in lambs is variable. According to studies by [34,35], supplementing with ZH for 30 d can increase the toughness of the LTM by 20.0 to 56.4%, which is consistent with our research showing a 21.5% increase in WBSF. However, other reports by [34,47] have not found any differences in WBSF between lambs treated with ZH and the control group.

The final pH of the meat is a crucial factor in determining its quality, and lambs supplemented with ZH have been observed to produce meat with an elevated pH of 3.2 to 8.9% [31]. Our experiment’s results align with this last result. The findings on the color of lamb meat, when supplemented with ZH, are inconsistent. According to research [34,48], there were no discernible differences in the luminosity (L*), redness (a*), and yellowness (b*) color characteristics of the LTM after administering ZH for 28–30 d. However, other studies have reported a decrease in luminosity (L*) and redness (a*) by up to 13.8% and 24.2%, respectively. In the current experiment, ZH only resulted in a 7.0% decrease in luminosity without any impact on the redness of the meat.

A higher concentration of calcium in muscles is decisive for the function of this system, which is key to determining the postmortem quality characteristics of meat tenderness [20]. The proteolytic system of the calpain–calpastatin muscle system is a calcium-dependent enzymatic system. Therefore, it increases the activity of calpain enzymes that degrade myofibrillar proteins [20] and promotes meat tenderness [17,18,19]. Vitamin D_3_ supplemented before slaughter increases the concentration of intramuscular calcium [16,17,18]; therefore, it could increase the activity of the calpain enzyme system and promote meat tenderness [15]. Studies have shown that D3 supplementation can indeed increase meat tenderness [16,17,18]. In this regard, studies on cattle, with D3 doses ranging from 1 to 7 million IU, have been shown to reduce the WBSF of the LTM [18,40]. They used supra-nutritional doses of D3 in the diet for a period ranging from 3 to 7 d before slaughter. Other authors [8,22] have reported that supplementing with 0.5–5 × 10^6^ IU/d of D3 between 10 and 20 d before slaughter did not affect the WBSF values of meat, even with 14 to 35 d of aging. However, [22] found that a treatment of 0.25 × 10^6^ IU/d with D3 for 165 d before slaughter tended to produce tougher meat at 21 d of aging. The findings align with those reported by [41] in their study on lambs supplemented with 750,000 IU/d of D3 for 4 d, which resulted in meat with 11.4% toughness. However, in the current experiment, no significant difference was observed in the WBSF of muscle samples collected from animals supplemented with D3 for 7 d before slaughter.

The water content of meat products is one of the essential quality characteristics; it also has a substantial influence on product quality, as a higher loss of water gives an expectation of a less optimal quality, which, if excessive, can have an adverse effect on product appearance [49]. Studies with lambs supplemented with ZH showed increased water purge loss of meat between 20 and 44.0% [34,50]. Our experiment showed similar results; the WHC of lamb meat was reduced with ZH supplementation. Likewise, in the current study, meat samples from lambs treated with ZH (Table 6) showed higher pH values and, conversely, luminosity (L*) and red intensity (a*) were lower (Figure 5). Higher pH values in meat are associated with the supply of β-agonists, which may be due to lower deposition of muscle glycogen, affecting pH drops [47].

If the final pH of the meat is high, the physical state of the proteins will be above their isoelectric point. Proteins will associate with more water in the muscle and, therefore, the fibers will be tight (less space between cells). This meat will appear to have lower luminosity (L*) due to the fact that its surface does not scatter light to the same extent as the more open surface of the meat with a lower final pH [51]. The oxygen penetration depth and formation of oxymyoglobin (red myoglobin form) is dependent upon the partial pressure of oxygen and its ability to diffuse into the muscle structure. It is possible that changes in the structure and spacing of the muscle could alter this depth and, hence, the oxygen consumption rate. In high-pH dark muscle, the lack of spaces between cells and muscle bundles prevents oxygen diffusion into the tissue, and there is a greater demand for oxygen by myoglobin in the interior; hence, at the surface, there is less oxymyoglobin, which is observed as a reduction in redness (a*) values [52].

Different studies have implemented D3 to mitigate the negative impact of meat toughness caused by ZH supplementation. Authors [18,22] have reported slight improvements in beef tenderness in cattle that were supplemented with ZH and given D3 at doses of 1 to 7 million IU per d for 3 to 6 d. Other studies [46] in steers supplemented with 1–9 × 10^6^ IU between 3 and 9 d before slaughter did not reduce meat toughness produced by ZH supplementation. ZH supplementation resulted in tougher meat in 7.0–21.4% of the LTM on different aging periods, and the supplementation of 0.5 × 10^6^ IU D3 did not reduce the negative effect of zilpaterol. [8] In our experiment, supplementing with 1.5 × 10^6^ IU of D3 did not result in reduced WBSF values of the LTM of feedlot lambs with ZH supplementation, which is similar to the outcome mentioned earlier.

### 4.3. Fatty Acids

β-agonists have been studied for their effect on the fatty acid profile of ruminants. In a study by [3,47], lambs treated with ZH showed an increase in polyunsaturated fatty acids in intramuscular fat. In their study on lambs, [53] found that administering Salbutamol, a β-agonist, led to a decrease in saturated fatty acids and an increase in total unsaturated fatty acids. Nonetheless, the research conducted by [31] revealed that the addition of ZH to the diet of lambs did not result in any alteration to the fatty acid composition of the intramuscular fat. However, the findings of our current study demonstrated that supplementing lambs with ZH did not impact the overall levels of saturated or unsaturated fatty acids present in intramuscular fat but tended to reduce monounsaturated fatty acid (C16:1). The variation in the results obtained through the use of β-agonists in the fatty acid profile of meat can be attributed to various factors. These factors include genetic influence by breed, the type of β-adrenergic receptor that is stimulated (β_1_ or β_2_), and the concentration of β-adrenergic receptors that increase with age due to tissue development [54]. Additionally, the composition of fatty acids in meat can be affected by factors, including the degree of fattening and the composition of the diet consumed by ruminants [55].

Research with D3 in ruminants has focused on calcium metabolism and its effect on meat tenderness. Thus, research on the use of D3 in muscle fatty acid metabolism is scarce. In this sense, the fatty acid profile of meat is better related to vitamin E supplementation because of the oxidative stability of fats [15]. However, studies in mice have shown that D3 deficiency plays an important role in the metabolism of fatty acids in the blood and liver [56]. In addition, it has been observed that supplementation of D3 also stimulated the expression of genes promoting fatty acid oxidation, such as carnitine palmitoyl-transferase I α or β, medium- and long-chain acyl-CoA dehydrogenases, as well as the expression of the pyruvate dehydrogenase kinase 4 mRNA, which is known to encode an enzyme that favors the utilization of fatty acids over glucose [57].

However, in the present experiment, it is observed (Figure 8) that D3 supplementation results in a reduction in oleic fatty acid. Likewise, an interaction was observed, and a change in the metabolism of PUFAs and UFAs in intramuscular fat was noted when the lambs were supplemented with ZIL+VIT. The reason for this interaction is not clear. Probably, the negative effect of DMI by D3 produces this response, which may be altered by the lipid metabolism of ZH in muscle.

## 5. Conclusions

Supplementation with ZH for 26 d plus a 3 d withdrawal period improved growth performance, energy efficiency, and carcass characteristics. However, it negatively affected meat quality by reducing lightness and increasing pH and WBSF. Supplementation with D3 for 7 d before slaughter had a negative impact on the growth performance and energy efficiency. This was due to a reduction in DMI during the last 7 d of the trial. Additionally, D3 seemed to reduce HCW, although no differences in terms of dressing carcass and LTM area were noted. Administering D3 at a dose of 1.5 × 10^6^ IU/^−1^ for 7 d prior to slaughter in lambs treated with ZH improved meat quality by increasing redness and reducing muscle pH compared to lambs treated with ZH alone. However, D3 did not improve the meat tenderness negatively affected by ZH supplementation.

## Figures and Tables

**Table 1:** The table shows the formula of the experimental diets.

Ingredients	Inclusion, %
Sudangrass hay	14.5
Cracked corn	66.0
Soybean meal	6.0
Grease trap waste	2.0
Molasses cane	8.0
Urea	1.0
Trace mineral salt	2.5
Nutritional composition ^a^	
Dry matter, %	89.0
Crude protein, %	14.5
NE_m_, Mcal/kg/Dry matter	2.06
NE_g_, Mcal/kg/Dry matter	1.40

^a^ Derived from tabular values based on ingredient composition of the experimental diet.

**Table 2:** The table shows the main effects of zilpaterol and vitamin D_3_ on the growth performance of feedlot lambs.

	Treatments ^a^							
	ZH		D3			*p*-Value		
Variables	0	0.20	0	1.5	SEM	ZH	D3	ZH×D3
Live weight								
Initial	37.32	37.32	37.20	37.44	0.261	1.00	0.53	0.81
Final	44.39	45.09	45.36	44.12	0.369	0.21	0.04	0.12
DMI								
Daily, g	1240	1180	1180	1230	0.027	0.13	0.24	0.57
As % of LW	3.06	2.86	2.88	3.04	0.05	0.04	0.08	0.28
Last 7 d, g/d	1330	1260	1400	1180	0.05	0.41	0.02	0.62
Weight gain								
Daily, g	243	267	281	230	0.007	0.05	<0.01	0.01
Total, kg	7.07	7.76	8.15	6.68	0.222	0.05	<0.01	0.01
Feed efficiency								
DMI/Gain	5.14	4.54	4.28	5.39	0.166	0.03	<0.01	0.03
Gain/DMI	197	232	242	187	0.007	0.01	<0.01	0.01
Adjusted Carcass ^b^								
FLW	43.90	45.78	45.43	44.25	0.409	0.01	0.07	0.07
ADG, g	227	291	283	235	0.009	<0.01	<0.01	<0.01
DMI/ADG	5.76	4.24	4.43	5.56	0.210	<0.01	<0.01	<0.01
ADG/DMI	182	249	242	189	0.008	<0.01	<0.01	<0.01

^a^ ZH, Zilpaterol hydrochloride, (0 and 0.20 mg/kg LW^−1^) 26 d of supplementation plus 3 d of withdrawal; D3, Vitamin D_3_, (0 and 1.5 × 10^6^ IU/lamb/d^−1^) 7 d of supplementation plus 1 d of withdrawal. ^b^ Growth performance components calculated from FLW-adjusted carcass.

**Table 3:** The table shows the main effects of zilpaterol and vitamin D_3_ on the dietary energetics of feedlot lambs.

	Treatments ^a^							
	ZH		D3			*p*-Value		
Variables	0	0.20	0	1.5	SEM	ZH	D3	ZH×D3
Net Energy								
NEm Mcal/Kg/DM	2.02	2.25	2.30	1.96	0.047	<0.01	<0.01	0.01
NEg Mcal/Kg/DM	1.36	1.56	1.61	1.31	0.042	<0.01	<0.01	0.01
Obs/exp DMI ^b^	1.06	0.94	0.90	1.08	0.025	0.01	<0.01	0.02
Obs/exp NE ^c^								
Maintenance	0.96	1.07	1.09	0.93	0.022	<0.01	<0.01	0.01
Gain	0.95	1.09	1.12	0.92	0.029	<0.01	<0.01	0.01

^a^ ZH, Zilpaterol hydrochloride, (0 and 0.20 mg/kg LW^−1^) 26 d of supplementation plus 3 d of withdrawal; D3, Vitamin D_3_, (0 and 1.5 × 10^6^ IU/lamb/d^−1^) 7 d of supplementation plus 1 d of withdrawal. ^b^ Observed/expected ratio of DMI. ^c^ Observed/expected ratio of net energy.

**Table 4:** The table shows the main effects of zilpaterol and vitamin D_3_ on the carcass traits of feedlot lambs.

	Treatments ^a^							
	ZH		D3			*p*-Value		
Variables	0	0.20	0	1.5	SEM	ZH	D3	ZH×D3
HCW, kg	24.40	25.45	25.25	24.60	0.227	0.01	0.07	0.07
Dressing, %	54.85	56.31	55.53	55.62	0.248	<0.01	0.80	0.39
LTM area, cm^2^	13.56	14.47	14.20	13.83	0.254	0.03	0.32	0.95
Fat thickness, mm	2.60	2.44	2.46	2.58	0.084	0.21	0.32	0.75
Perirenal-pelvis fat, %	2.87	2.66	2.86	2.67	0.176	0.43	0.46	0.78

^a^ ZH, Zilpaterol hydrochloride, (0 and 0.20 mg/kg LW^−1^) 26 d of supplementation plus 3 d of withdrawal; D3, Vitamin D_3_, (0 and 1.5 × 10^6^ IU/lamb/d^−1^) 7 d of supplementation plus 1 d of withdrawal.

**Table 5:** The table shows the main effects of zilpaterol and vitamin D_3_ on primary cuts of feedlot lambs.

	Treatments ^a^							
	ZH		D3			*p*-Value		
Primary Cuts ^b^	0	0.20	0	1.5	SEM	ZH	D3	ZH×D3
Neck ^c^	5.14	4.97	4.99	5.12	0.147	0.49	0.57	0.13
Foreshank-208D	17.7	17.6	17.7	17.5	0.160	0.80	0.42	0.67
Rib-209A	7.96	8.15	8.03	8.08	0.121	0.49	0.87	0.81
Rack-204	9.22	9.46	9.39	9.29	0.091	0.27	0.62	0.92
Breast-209	4.12	3.99	4.16	3.94	0.175	0.75	0.59	0.89
Shoulder-207	8.96	9.27	9.02	9.22	0.252	0.60	0.74	0.90
Flank-232E	6.15	6.24	6.30	6.08	0.094	0.65	0.27	0.40
Loin-232A	9.47	9.5	9.45	9.53	0.115	0.91	0.80	0.98
Leg-233A	31.2	30.7	30.8	31.1	0.293	0.35	0.47	0.33

^a^ ZH, Zilpaterol hydrochloride, (0 and 0.20 mg/kg LW^−1^) 26 d of supplementation plus 3 d of withdrawal; D3, Vitamin D_3_, (0 and 1.5 × 10^6^ IU/lamb/d^−1^) 7 d of supplementation plus 1 d of withdrawal. ^b^ NAMP, North American Meat Processors Association number item. ^c^ Not included in NAMP’s list as a primary cut.

**Table 6:** The table shows the main effects of zilpaterol and vitamin D_3_ on the meat quality characteristics of feedlot lambs.

	Treatments ^a^							
	ZH		D3			*p*-Value		
Variables	0	0.20	0	1.5	SEM	ZH	D3	ZH×D3
Color ^b^								
L*	33.33	30.99	30.92	33.41	0.683	0.02	0.01	0.54
a*	16.37	15.80	15.56	16.61	0.540	0.16	0.01	<0.01
b*	14.08	13.17	12.55	14.69	0.644	0.32	0.02	0.20
pH	5.59	5.73	5.71	5.61	0.032	<0.01	0.03	<0.01
WBSF ^c^, kg	2.39	2.94	2.62	2.71	0.090	<0.01	0.43	0.09
WHC ^d^, %	87.23	84.18	86.97	84.44	0.540	<0.01	<0.01	0.91

^a^ ZH, Zilpaterol hydrochloride, (0 and 0.20 mg/kg LW^−1^) 26 d of supplementation plus 3 d of withdrawal; D3, Vitamin D_3_, (0 and 1.5 × 10^6^ IU/lamb/d^−1^) 7 d of supplementation plus 1 d of withdrawal. ^b^ L*, Lightness; a*, Redness; b*, Yellowness. ^c^ WBSF, Warner–Bratzler shear force. ^d^ WHC, Water holding capacity.

**Table 7:** The table shows the main effects of zilpaterol and vitamin D_3_ on the fatty acids profile of meat of feedlot lambs.

	Treatments ^a^							
	ZH		D3			*p*-Value		
Fatty Acid	0	0.20	0	1.5	SEM	ZH	D3	ZH×D3
C14:0 Myristic	4.13	3.03	3.59	3.56	0.511	0.15	0.97	0.55
C14:1 Myristoleic	0.30	0.20	0.25	0.25	0.056	0.22	0.93	0.30
C16:0 Palmitic	31.04	29.70	29.42	31.32	0.779	0.24	0.11	0.29
C16:1 Palmitoleic	2.31	1.86	2.09	2.08	0.180	0.10	0.96	0.45
C18:0 Stearic	11.41	12.60	11.96	12.04	0.567	0.16	0.92	0.44
C18:1 Oleic	46.29	47.88	48.49	45.68	1.120	0.33	0.10	0.03
C18:2 Linoleic	4.12	4.48	3.90	4.70	0.710	0.72	0.44	0.30
C18:3 Linolenic	0.22	0.18	0.27	0.13	0.044	0.46	0.05	0.96
C20:0 Arachidonic	0.14	0.04	0.01	0.19	0.108	0.50	0.23	0.50
Saturated	46.58	45.34	44.98	46.94	0.901	0.34	0.15	0.10
Unsaturated	53.41	54.65	55.01	53.05	0.901	0.34	0.15	0.10
Unsat/Satur	1.15	1.21	1.23	1.13	0.041	0.39	0.14	0.09

^a^ ZH, Zilpaterol hydrochloride, (0 and 0.20 mg/kg LW^−1^) 26 d of supplementation plus 3 d of withdrawal; D3, Vitamin D_3_, (0 and 1.5 × 10^6^ IU/lamb/d^−1^) 7 d of supplementation plus 1 d of withdrawal.

## Data Availability

The information published in this study is available upon request from the corresponding author.
